# Comparative single-cell landscape of immune cells in human livers affected HBV and non-viral cirrhosis

**DOI:** 10.3389/fmed.2025.1708675

**Published:** 2026-02-20

**Authors:** Qingquan Bai, Zhengyang Zhao, Jiashu Zou, Chenhao Wang, Xiao He, Shuqi Mao, Yongcheng Su, Da Li, Bowen Zheng, Shengdong Wu, Caide Lu, Meiya Chen

**Affiliations:** 1 Department of Gastroenterology, The National Key Clinical Specialty, Zhongshan Hospital of Xiamen University, School of Medicine, Xiamen University, Xiamen, Fujian, China; 2 Department of Hepatobiliary and Pancreatic Surgery, Ningbo Medical Centre Lihuili Hospital, Affiliated Lihuili Hospital of Ningbo University, Ningbo, China; 3 Department of Hepatology and Gastroenterology, Charité Universitätsmedizin Berlin, Berlin, Germany; 4 Cancer Institute, Shenzhen Peking University-the Hong Kong University of Science and Technology (PKU-HKUST) Medical Center, Shenzhen, China; 5 Institute of Cancer Research, Shenzhen Bay Laboratory, Shenzhen, China; 6 Department of Hepatic Surgery, The First Affiliated Hospital of Harbin Medical University, Harbin, China; 7 Army 73rd Group Military Hospital, Xiamen, China; 8 Department of Neurology, Ningbo Medical Treatment Center Lihuili Hospital, Ningbo, China; 9 Department of Breast Surgery, The First Hospital of Xiamen University, School of Medicine, Xiamen University, Xiamen, China; 10 Department of Gastrology, The First Affiliated Hospital of Tsinghua University, Beijing, China; 11 Cancer Research Center, School of Medicine, Xiamen University, Xiamen, China; 12 Clinical Research Center for Gut Microbiota and Digestive Diseases of Fujian Province, Xiamen Key Laboratory of Intestinal Microbiome and Human Health, Xiamen, China; 13 The School of Clinical Medicine, Fujian Medical University, Fuzhou, China

**Keywords:** cirrhosis, HBV cirrhosis, immune cell landscapes, non-viral cirrhosis, ScRNA-seq

## Abstract

**Background:**

Cirrhosis, particularly HBV-induced, poses a significant global health burden. This study compares immune cell landscapes in HBV and non-viral cirrhosis using single-cell RNA sequencing (scRNA-seq) to elucidate distinct immune mechanisms driving disease progression.

**Methods:**

Liver tissues from HBV cirrhosis patients and healthy controls were analyzed via scRNA-seq. Public single-cell and spatial transcriptomics data were integrated to map immune cell populations. Computational analyses included differential expression, pathway enrichment (KEGG/GO), and cell–cell communication (CellChat).

**Results:**

HBV cirrhosis exhibited expanded Macrophage-PLCG2 (anti-inflammatory) and CD8 + T-FABP5 subsets, while Macrophage-CD5L, CD4 + T-ANXA1, CD4 + T-CCR6, and NK-FCER1G were reduced. Pathway analysis linked Macrophage-PLCG2 to Rap1 signaling, whereas Macrophage-CD5L associated with lysosomal pathways. Spatial analysis revealed myeloid-T cell colocalization in HBV cirrhosis. Enhanced HLA-E/KLRK1 signaling between myeloid and NK cells was identified in HBV cases (*p* < 0.05).

**Conclusion:**

This study delineates immune cell heterogeneity between HBV and non-viral cirrhosis, highlighting potential therapeutic targets like Macrophage-PLCG2 and CD8 + T-FABP5. Findings underscore the role of immune dysregulation in HBV cirrhosis progression.

## Introduction

Cirrhosis is a common condition globally and is linked to significant incidence and mortality rates; each year around 2 million people die from liver diseases worldwide, with cirrhosis accounting for 1 million of these deaths (1). Causes of cirrhosis include alcohol-related liver disease (ALD) (2), non-alcoholic fatty liver disease (NAFLD) (3), primary biliary cirrhosis (PBC), hepatitis B virus (HBV) and hepatitis C virus. HBV, as a hepatotropic virus, can induce liver cell damage, immune cell infiltration, and liver fibrosis, ultimately leading to HBV cirrhosis (4).

The immune pathogenesis of HBV-related cirrhosis involves complex virus-host interactions, characterized by dysregulated T-cell responses and altered innate immune cell functions (5, 6). In contrast, non-viral cirrhosis, such as ALD and NAFLD, is driven by sterile inflammation mediated by metabolic stress and DAMPs (7–9). Previous studies have suggested distinct immune profiles between viral and non-viral etiologies (10–13), yet a comprehensive understanding at the single-cell level is lacking.

With the advancement of high-throughput sequencing, particularly the emergence of single-cell omics (14), we have gained deeper insights into the cellular heterogeneity of diseases, classification, and the discovery of immune cell subpopulations. Recent scRNA-seq studies have revealed immune heterogeneity in liver fibrosis (15, 16), but a direct comparison between HBV and non-viral cirrhosis remains unexplored.

To our knowledge, this study represents integrated single-cell transcriptomic analyses to specifically compare the immune landscapes of HBV-related and non-viral cirrhosis.

## Materials and methods

### Human sample collection

Liver tissues were approved by the Ethics Review Center of the First Affiliated Hospital of Harbin Medical University (Approval No. 2021179). The cohort included two healthy donors and two patients with hepatitis B virus (HBV) cirrhosis (Supplementary Table S1). Informed consent was obtained from all the patients and donors.

### Sample size and data integration

This study initially included a cohort of 2 patients with HBV-related cirrhosis and 2 healthy controls for scRNA-seq. To enhance the statistical power and robustness of our findings, we integrated our data with two publicly available single-cell datasets of human liver cirrhosis. We also obtained single-cell transcript transcriptome data of 5 healthy livers, 5 non-viral from PRJNA561303 (15) and 3 HBV-related cirrhotic livers from HRA000069 (17). The combined dataset comprised a total of 17 samples (7 healthy controls, 5 HBV cirrhosis, and 5 non-viral cirrhosis), yielding 94,127 high-quality immune cells for subsequent analysis. This integration strategy allowed us to analyze immune cell landscapes across a larger and more diverse patient background, thereby mitigating the limitations associated with a small initial sample size and increasing the generalizability of our observations.

**Figure 1:**
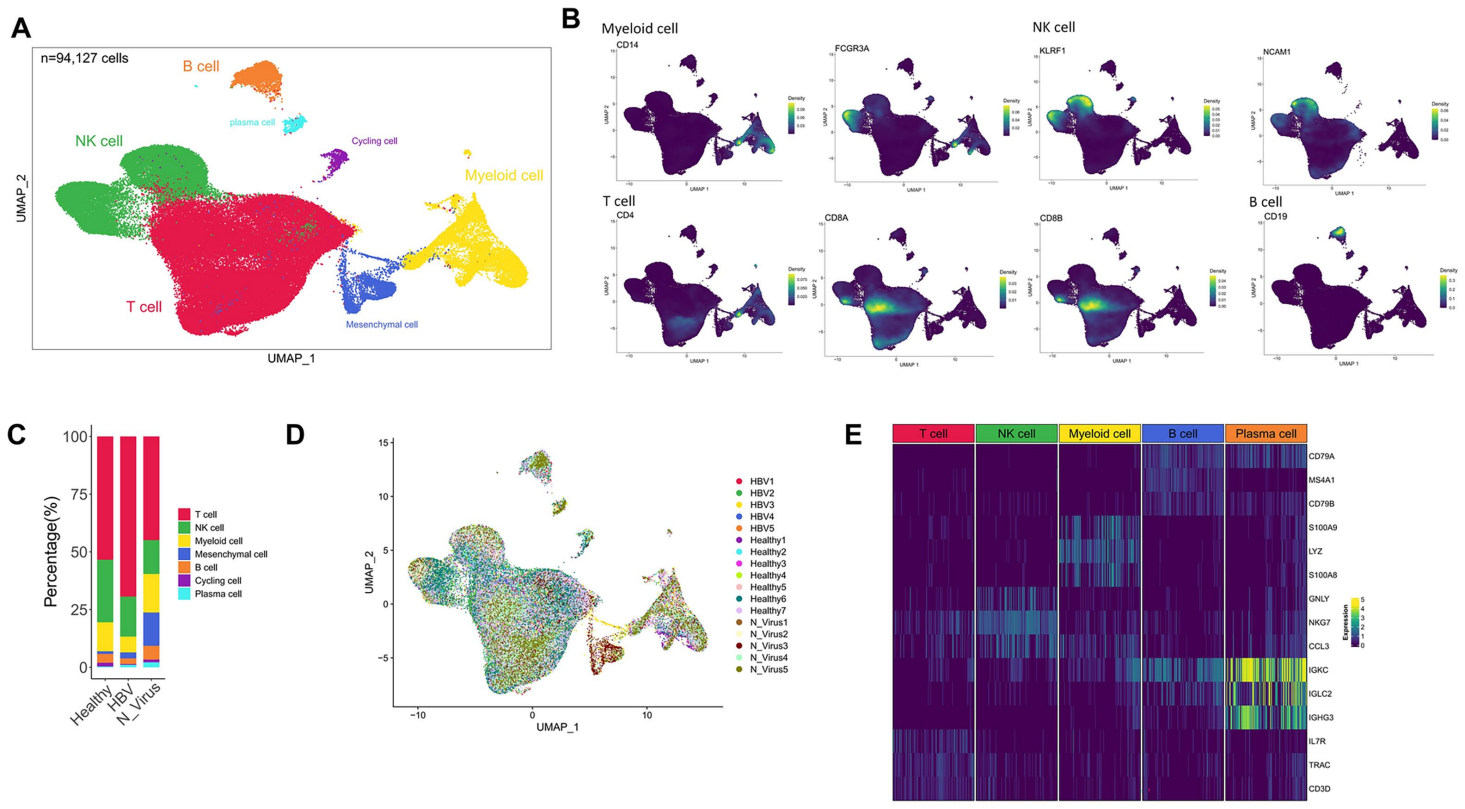
Single-cell atlas of HBV and non-viral cirrhosis and healthy liver. **(A)** Clustering analysis of all CD45 + leucocytes. **(B)** UMAP plot showing the expression of CD14, FCGR3A (CD16), KLRF1, NCAM1, CD4, CD8A, CD8B, and CD19. **(C)** The proportion of T cells, NK cells, myeloid cells, and B cells in healthy liver and HBV and non-viral cirrhosis samples. **(D)** UMAP plots showing the distribution of major cell clusters in healthy liver, HBV, and non-viral cirrhosis samples. **(E)** Gene expression heatmap in each cell cluster. NK, natural killer; UMAP, uniform manifold approximation and projection.

During the data quality control process, we screened for high-quality cells through rigorous quality assessment criteria: cells with the number of detected genes < 200 or > 6,000 were excluded, cells with a mitochondrial gene proportion > 20% were removed. To address potential batch effects introduced by combining datasets generated from different studies and platforms, we utilized the integration method implemented in the Seurat package. Specifically, we identified integration anchors across datasets using the FindIntegrationAnchorsfunction and then integrated the data using the IntegrateDatafunction. This approach effectively corrected technical variations, as evidenced by the uniform distribution of cells from different sources in the UMAP projection (Figure 1D), without clustering by batch.

### Human tissue processing

Liver tissues from surgery were quickly diluted in pre-cooled DMEM medium and transferred to a sterile lab. After triple washing with PBS, tissues were diced into 1–3 mm^3^ pieces, mixed with a cryopreservation solution (10% DMSO and 90% fetal bovine serum), and stored at −80 °C. Frozen tissue samples were taken out of the −80 °C refrigerator and immediately placed in a 37 °C water bath for thawing until ice crystals in the samples completely melted. After thawing, the samples were washed twice with DMEM medium to remove residual DMSO. The trypan blue staining method was used, and only samples with a cell viability of more than 90% were used for subsequent experiments.

### Droplet-based single-cell RNA sequencing

Tissues were converted to single-cell suspensions utilizing a multi-tissue dissociation kit. Haematopoietic cells were isolated by staining with PerCP-Cyanine5.5 conjugated CD45 monoclonal antibody and propidium iodide dye.

### Processing of scRNA-seq

We used Cellranger for filtering, genome alignment, and generation of HDF5 matrix files. Subsequently, we imported these files into Seurat (V4.3.0) for processing according to its standard single-cell workflow. Batch effects were mitigated using Seurat’s integration anchors.

### Processing of spatial transcriptomics sequencing (ST-seq)

The standard workflow for analyzing 10x Visium spatial transcriptomics data using Seurat included normalization, identification of highly variable genes, and scaling using the SCTransform function.

### Enrichment analysis

We identified differentially expressed genes (DEGs) in immune cell subpopulations using the FindAllMarkers filtering with *p* < 0.05. Gene ID conversion was performed using the org.Hs.eg.db (v 3.16.0) database, followed by KEGG and GO enrichment analysis using ClusterProfiler (v 4.6.2).

### Cell–cell communication

We utilized CellChat (18) version (v 2.1.2) to investigate the differences in immune cell communication between HBV and non-virus patients. Networks with fewer than 10 cells were filtered out to ensure meaningful cell–cell communication analysis.

### Analysis of transcription factor regulatory networks

To conduct an analysis using SCENIC (19) (v 1.3.1), the required files include the transcription factor list hs_hgnc_tfs.txt, which can be downloaded from (https://github.com/aertslab/pySCENIC/blob/master/resources/hs_hgnc_tfs.txt). Additionally, the gene-motif ranking files needed for annotating each motif with the corresponding transcription factor can be downloaded from (https://resources.aertslab.org/cistarget/).

### Estimation of cell-type abundance in spatial transcriptomics spots

We employed Cell2location (20) (v 0.0.6a) to estimate the cell type composition of each spatial transcriptomics spot. We processed according to Cell2location’s recommended workflow: set max_epochs = 30,000 and N_cells_per_location = 30.

### Immune cells co-localization

We used Mistyr (21) (v1.6.1) to investigate the co-localization of immune cells in cirrhosis. Since each spot in the 10x Visium spatial transcriptomics data has a diameter of 55 μm, and immune cells typically range from 6 to 15 μm in size, while macrophages range from 15 to 30 μm, we configured the tool to view the distribution of immune cells within each spot.

### Immunofluorescence

Liver sections were baked at 63 °C for 2 h, then deparaffinized with xylene and treated with decreasing concentrations of ethanol. Antigen retrieval was performed by heating in DAKO antigen retrieval solution at 95 °C for 10 min. Sections were blocked in 10% goat serum in PBS for 20 min at room temperature (RT), washed, and incubated with primary antibodies (CD5L Polyclonal antibody, 17,224-1-AP, Proteintech, Wuhanm, China; PLCG 2 Recombinant Rabbit Monoclonal Antibody, HA721477, HUABIO, Hangzhou, China) diluted in blocking solution at 4 °C overnight. They were then incubated with Cy3- and FITC-conjugated secondary antibodies for 30 min at room temperature, stained with DAPI for 10 min at RT, and mounted with VECTASHIELD to prevent photobleaching.

## Results

### Single-cell atlas of immune cells in healthy, HBV, and non-viral cirrhosis

Single-cell transcriptome data were obtained from 45,993 immune cells. After integration with datasets from two prior studies (15, 17) utilizing Seurat, a total of 94,127 immune cells were analyzed from 7 healthy liver samples, 5 HBV cirrhosis patient samples, and 5 non-viral cirrhosis patient samples.

After dimensionality reduction using PCA and UMAP, cell clustering based on KNN identified 23 cell subgroups and these cells were classified into five major cell subtypes (Figure 1A). The subtypes included T cells, which expressed CD4, CD8A, and CD8B; myeloid cells, which expressed CD14 and FCGR3A; NK cells, which expressed KLRF1 and NCAM1; B cells, which expressed CD19; and plasma cells, identified as PC-1 (Figure 1B). Figure 1C compares the proportion of these five main cell subtypes in healthy, HBV, and non-viral cirrhosis. The integration of multiple datasets significantly expanded the sample size, allowing for a better comparison. The effectiveness of batch effect correction is demonstrated by the intermingled distribution of cells from different studies in the UMAP plot, rather than separation by dataset origin (Figure 1D). Nonetheless, we acknowledge that inherent technical differences between studies remain a potential limitation, and conclusions would benefit from future validation in larger, independent cohorts. Figure 1E depicts the marker genes in the subclusters. We also identified contamination of non-hematopoietic cells, including cycling cells and mesenchymal cells, and the rate of contamination was found to be low, accounting for only 4.94% of the total number of cells.

### Macrophage-CD5L expanded in non-viral cirrhosis and macrophage-PLCG2 expanded in HBV cirrhosis

**Figure 2:**
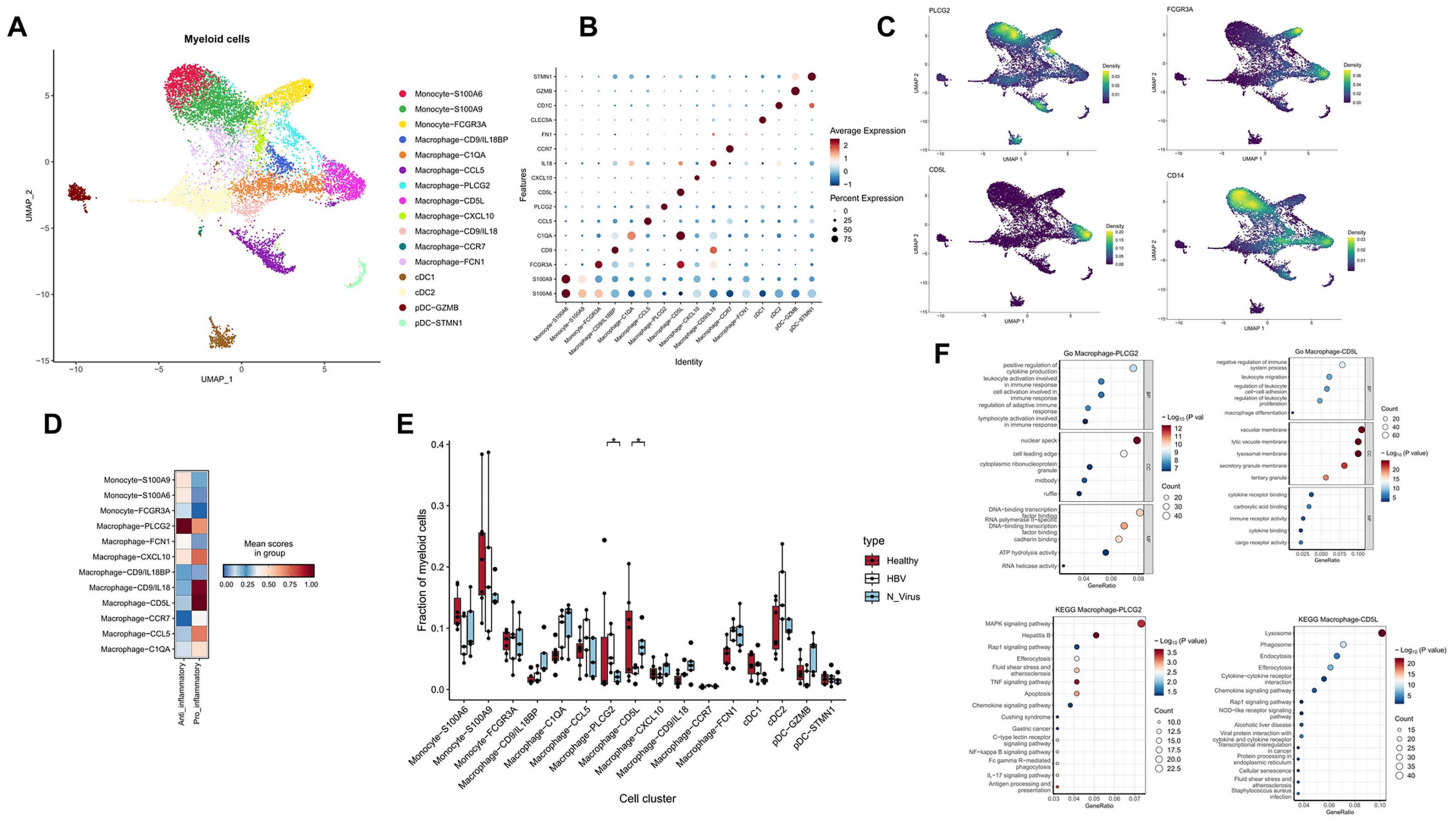
The subtypes of myeloid cells in healthy liver and HBV and non-viral cirrhosis. **(A)** Clustering analysis of all myeloid cells. **(B)** Dot plot illustrating the expression of marker genes in each cluster. **(C)** The expression of PLCG2, FCGR3A (CD16), CD5L, and CD14 in the UMAP plot. **(D)** Pro-inflammatory and anti-inflammatory scores between healthy liver and HBV and non-viral cirrhosis in clusters of macrophages and monocytes. **(E)** The proportion of different clusters in myeloid cells in healthy liver and HBV and non-viral cirrhosis. *p < 0.05 (Mann–Whitney U test). **(F)** Gene ntology (GO) and Kyoto ncyclopedia of enes and enomes (KEGG) enrichment analysis of Macrophage-PLCG2 and Macrophage-CD5L marker genes.

We further categorized the myeloid cell population into 16 distinct subgroups (Figures 2A,B). The relative proportions of these myeloid cell subclusters were analyzed and compared between non-virus and HBV cirrhosis cases (Figures 2C–F).

Three specific monocyte subclusters were identified based on their key marker genes (Figures 2A,B). The subclusters Monocyte-S100A9 and Monocyte-S100A6 were identified as CD14 + classical monocytes marked by elevated levels of S100A4, S100A6, S100A9, and S100A11 (Figure 2C). These classical monocytes are characterized by their activation state and readiness for phagocytosis (22). The Monocyte-FCGR3A subcluster was classified as nonclassical CD16 + monocytes due to the high expression of FCGR3A (CD16) (Figure 2C). Additionally, macrophages were divided into nine subpopulations according to the expression profiles of their characteristic marker genes (Figures 2A,B). To investigate the inflammatory states of macrophage subpopulations, we collected pro-inflammatory and anti-inflammatory gene sets and scored them using AddModuleScore. We found that the Macrophage-PLCG2 and CD14 + classical monocyte subpopulations (Monocyte-S100A9 and Monocyte-S100A6) exhibited a stronger anti-inflammatory tendency. In contrast, the Macrophage-CD9/IL8, Macrophage-CD5L, and Macrophage-CCL5 subpopulations showed more pronounced pro-inflammatory effects (Figure 2D).

We found that the proportion of Macrophage-PLCG2 is higher in HBV cirrhosis compared to non-viral cirrhosis in humans (*p* < 0.05) (Figure 2E). The PLCG2 protein is a transmembrane signaling enzyme critical for transmitting signals. Mutations in PLCG2 have been identified in autoimmune inflammation, antibody deficiencies, immune dysregulation syndrome, and familial cold autoinflammatory syndrome (23). Conversely, there is an increased proportion of Macrophage-CD5L in non-viral cirrhosis (*p* < 0.05) (Figure 2E). CD5L is a protein primarily expressed and secreted by macrophages, acting as an anti-apoptotic protein (24). Furthermore, Macrophage-CD5L is also marked by MARCO, leading us to consider Macrophage-CD5L as Kupffer cells.

KEGG enrichment analysis revealed that the Macrophage-PLCG2 phenotype is characterized by Hepatitis B, consistent with a higher proportion of Macrophage-PLCG2 in the HBV cirrhosis group. The Rap1 signaling pathway, related to cell-matrix adhesion, and pathways such as Efferocytosis, Apoptosis, IL-17, and NF-kappa B signaling play crucial roles in immune responses (Figure 2F). GO enrichment analysis indicated that this subset is also enriched in immune response regulation pathways. Additionally, it highlighted pathways related to gene expression and regulation, such as DNA-binding Transcription Factor Activity, as well as enzyme activity and metabolism pathways including ATP Hydrolysis Activity and RNA Helicase Activity.

**Figure 3:**
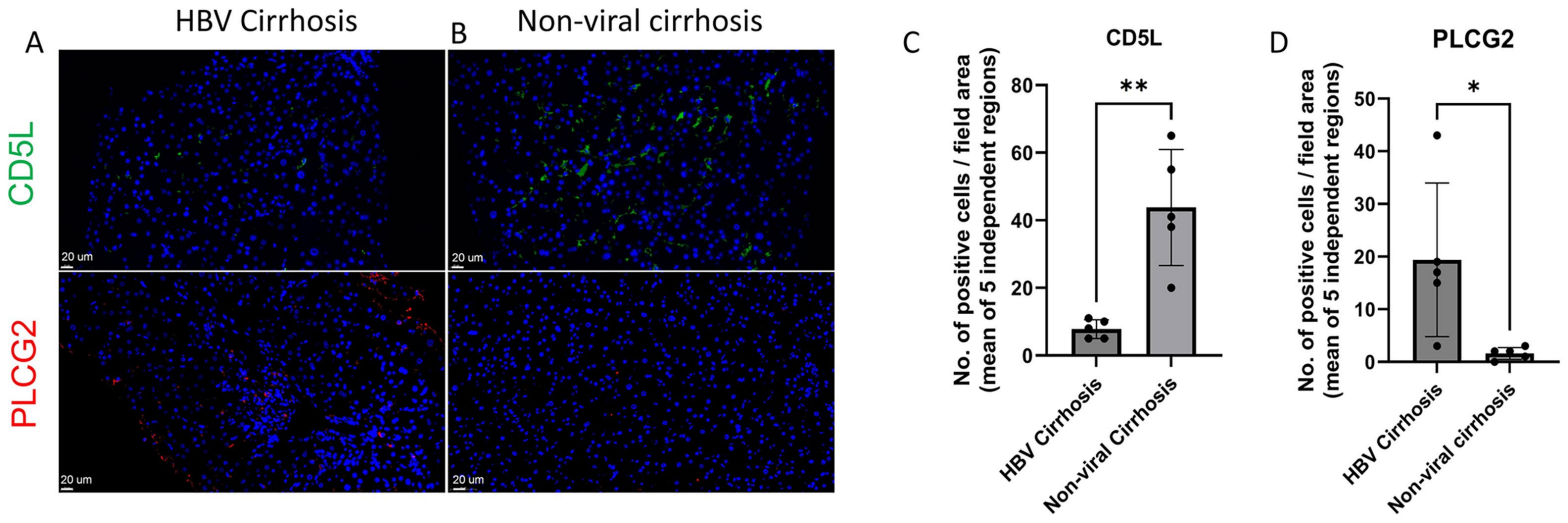
Immunofluorescence images showing CD5L and PLCG2 in **(A)** healthy liver, **(B)** HBV cirrhosis. **(C)** Quantification of CD5L positive cells from immunofluorescent images, * *p* < 0.01 (Mann–Whitney *U* test). **(D)** Quantification of PLCG2 positive cells from immunofluorescent images, *p < 0.05 (Mann–Whitney *U* test).

While KEGG enrichment analysis revealed that the Macrophage-CD5L subset is enriched in Lysosome, Phagosome, and Endocytosis pathways, and the Alcoholic Liver Disease Pathway. This aligns with the observation that Macrophage-CD5L is more prevalent in non-viral cirrhosis. Additionally, GO enrichment analysis indicated that this subset is enriched in pathways such as Negative Regulation of Immune System Process and Macrophage Differentiation (Figure 2F). Immunofluorescence staining validated the expansion of PLCG2-positive cells and the reduction in CD5L-positive cells in HBV-related cirrhosis compared with non-viral cirrhosis (Figures 3A–D).

### Transcription factor regulatory network analysis of myeloid cells in HBV and non-viral cirrhosis

**Figure 4:**
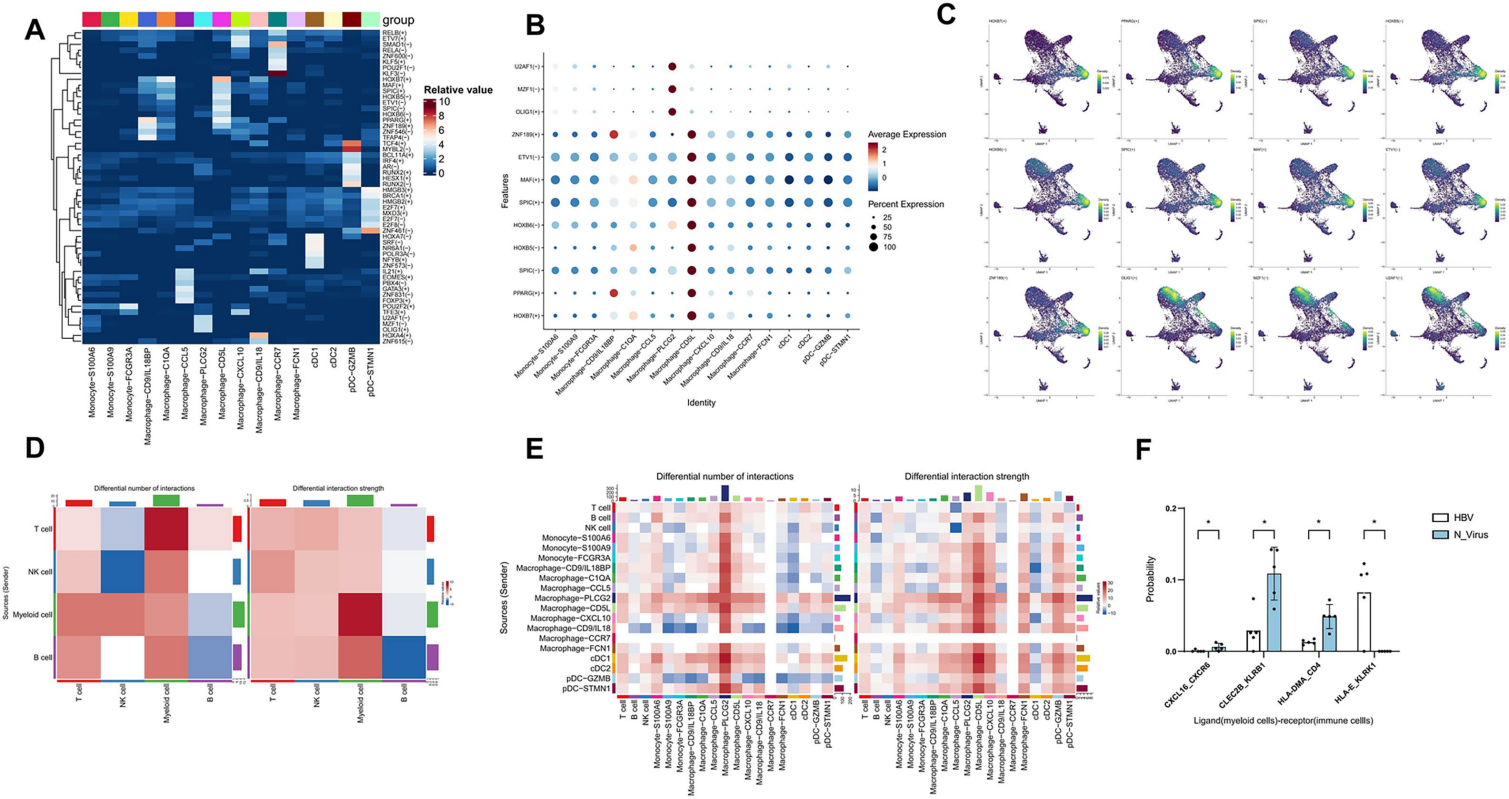
Transcription factor analysis and cell communication analysis of myeloid cells. **(A)** Heatmap displaying transcription factor activity of myeloid cells. **(B)** Dot plot illustrating transcription factor activity specific to the Macrophage-PLCG2 and Macrophage-CD5L subpopulations. **(C)** UMAP plots showing the distribution of transcription factors specific to the Macrophage-PLCG2 and Macrophage-CD5L subpopulations. **(D)** The number and strength of cell communication of immune cells in the HBV cirrhosis and non-viral cirrhosis. **(E)** The number and strength of cell communication of myeloid cell subclusters in the HBV cirrhosis and non-viral cirrhosis. **(F)** Cell communication of myeloid cell receptors and immune cell ligands. ^*^p < 0.05, ^**^p < 0.01, ***p < 0.001 (Wilcoxon test). In the color bar, red or blue indicates an increase or decrease in signal in HBV cirrhosis compared to non-viral cirrhosis.

To explore the functional differences within myeloid cell subpopulations, we conducted transcription factor (TF) analyses using SCENIC (Figure 4A), focusing on the subgroups Macrophage-CD5L and Macrophage-PLCG2, which exhibited notable differences between HBV-affected and non-virus livers (Figure 4B). We identified the main motifs for Macrophage-CD5L as HOXB7, HOXB5, and HOXB6, which are key in cell development and differentiation due to their roles in gene-encoded proteins. For Macrophage-PLCG2, the primary motifs were U2AF1, MZF1, and OLIG1. U2AF1 is previously established as a crucial RNA splicing factor. MZF1 inhibits T-cell infiltration, leading to resistance to immunotherapy in hepatocellular carcinoma (HCC) (25). Figure 4C shows the distribution of transcription factor scores in the Macrophage-CD5L and Macrophage-PLCG2 subgroups on a UMAP plot. However, the functions of these transcription factors in the context of cirrhosis remain to be elucidated.

### Enhanced HLA-E/KLRK1 signal pathway on myeloid cells and NK cells in HBV cirrhosis

We utilized CellChat to investigate the differences in cellular communication between HBV and non-viral cirrhosis. Notably, we observed that in the HBV group, compared to the non-virus group, In the HBV group, myeloid cells exhibit increased communication quantity and intensity both with T cells and NK cells, as well as within themselves. In contrast, communication quantity and intensity with B cells decreased (Figure 4D). In HBV, targeting Macrophage-PLCG2 subsets shows significantly enhanced cell communication intensity with other immune cell subsets (Figure 4E). Additionally, we found that KLRK1 receptors in NK cells were activated by Human Leukocyte Antigen E (HLA-E) in HBV cirrhosis (*p* < 0.05) (Figure 4F). HLA-E plays a crucial role in immune regulation through its interactions with NKG2A (encoded by KLRC1) and NKG2D (encoded by KLRK1) receptors. The interaction between HLA-E and NKG2A typically generates inhibitory signals that suppress the activity of NK cells and T cells, aiding tumors in evading immune surveillance (26). Conversely, the interaction between HLA-E and NKG2D promotes immune activation, enhancing the cytotoxic response of NK cells and T cells (27).

### NK-FCER1G expanded in non-viral cirrhosis

**Figure 5:**
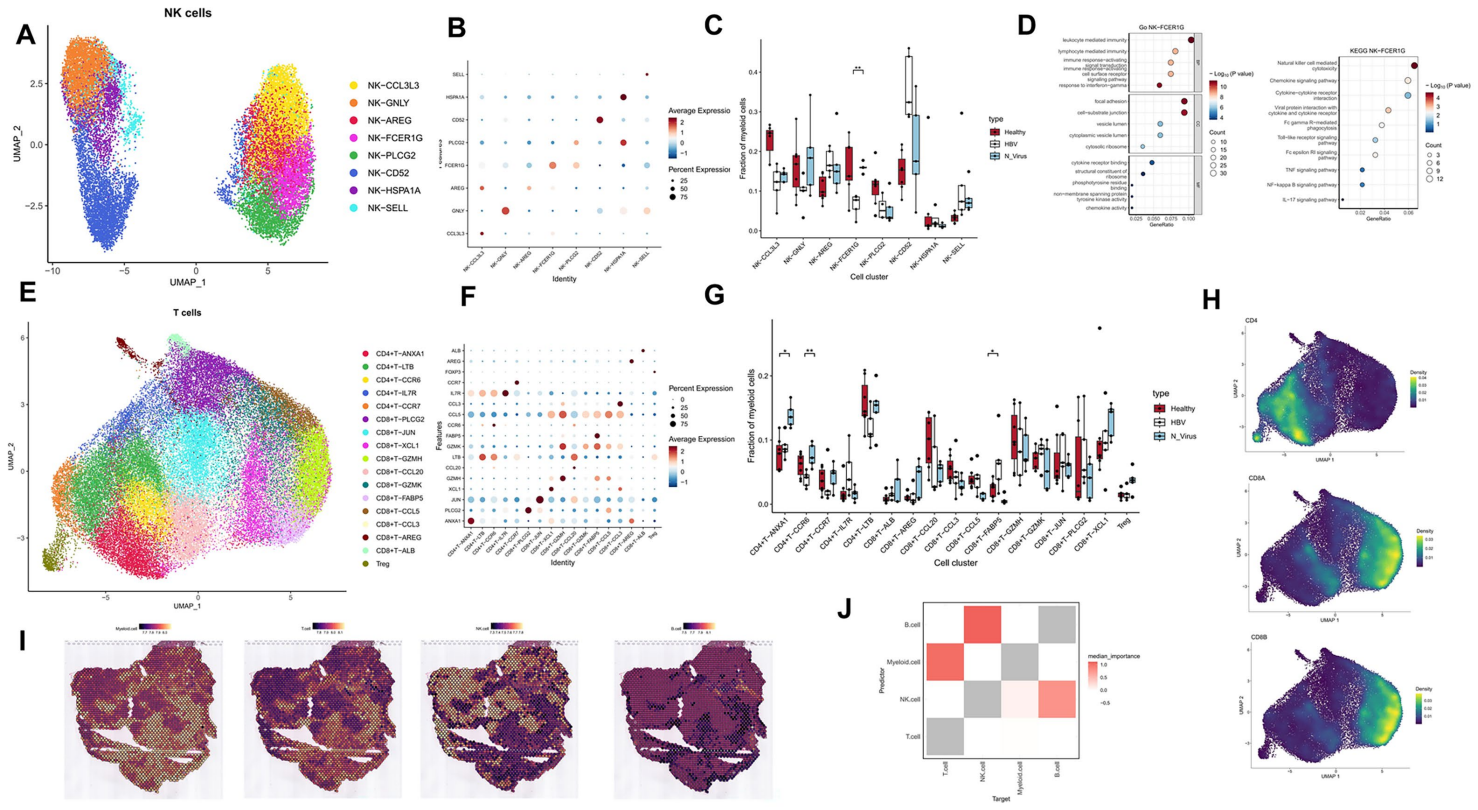
The subtypes of NK and T cells in healthy liver and HBV and non-viral cirrhosis samples and spatial distribution of immune cells. **(A)** Clustering analysis of NK cells. **(B)** Dot plot showing the marker genes in each cluster. **(C)** The proportion of NK cells subclusters in healthy liver and HBV and non-viral cirrhosis samples. *p < 0.05, **p < 0.01, ***p < 0.001 (Mann–Whitney *U* test). **(D)** GO and KEGG enrichment analysis of NK cell-FCER1G marker genes. **(E)** Clustering analysis of all the T cells. **(F)** Dot plot showing the marker genes in each cluster. **(G)** The proportion of T cell subclusters in healthy liver and HBV cirrhotic livers. **p < 0.01 (Mann–Whitney *U* test). **(H)** The expression of *CD4, CD8A,* and *CD8B* in UMAP plot. **(I)** Visualize the spatial distribution of immune cells in HBV cirrhosis tissue sections from HCC patients infected with HBV. **(J)** Heatmap illustrating the colocalization of immune cells in HBV cirrhosis.

We conducted an in-depth analysis of NK cells, identifying seven distinct subgroups (Figures 5A,B), and examined their proportions in healthy individuals, those with HBV, and non-viral cirrhosis (Figure 5C).

Based on marker genes, we identified the following NK cell subgroups: NK-CCL3L3, NK-GNLY, NK-AREG, NK-FCER1G, NK-PLCG2, NK-HSPA1A, and NK-SELL (Figures 5A,B). We observed that the proportion of NK-FCER1G was higher in non-viral cirrhosis than in HBV cirrhosis (*p* < 0.05) (Figure 5C). FCER1G, also known as FcRγ, is a protein expressed on the surface of immune cells such as eosinophils, dendritic cells, and NK cells, playing a crucial role in immune responses. It commonly binds to IgE and can also interact with IgG, participating in signal transduction, immune cell activation, antigen presentation, and other processes. Previous research has linked FCER1G to immune-related disorders like systemic lupus erythematosus and highlighted its importance in tumor immunotherapy (28). KEGG enrichment analysis of the NK-FCER1G subset revealed enrichment in pathways such as Natural Killer Cell Mediated Cytotoxicity and Fc Gamma R-Mediated Phagocytosis, where NK cells recognize and eliminate cells and pathogens via Fcγ receptors. The TNF Signaling Pathway was also enriched, indicating that NK cells can secrete TNF to exert antiviral effects. Additionally, GO enrichment analysis demonstrated the subset’s roles in immune regulation and cell migration (Figure 5D).

### CD4 + T-ANXA1 decrease and CD8 + T-FABP5 expanded in HBV cirrhosis

We conducted an analysis of T cell subsets comprising a total of 17 subgroups (Figures 5E,F). Based on marker genes, these subsets are CD4 + T-ANXA1, CD8 + T-PLCG2, CD8 + T-JUN, CD8 + T-XCL1, CD8 + T-GZMH, CD8 + T-CCL20, CD4 + T-LTB, CD8 + T-GZMK, CD8 + T-FABP5, CD4 + T-CCR6, CD8 + T-CCL5, CD8 + T-CCL3, CD4 + T-IL7R, CD4 + T-CCR7, Treg, CD8 + T-AREG, and CD8 + T-ALB (Figures 5E,F). We then compared the proportions of each T cell subset between non-virus and HBV cirrhosis (Figures 5G,H).

In HBV cirrhosis, there is a decrease in the proportion of CD4 + T-ANXA1. ANXA1, also known as lipocortin 1, is an intracellular calcium-binding protein. Several studies suggest that ANXA1 regulates T cell proliferation, differentiation, and cytokine production. ANXA1 also participates in regulating T cell migration, adhesion, and potentially exerts inhibitory effects. Additionally, ANXA1 can promote apoptosis of inflammatory cells and has been identified as one of the signals on apoptotic cells, enabling recognition and engulfment by phagocytic cells (29).

In HBV cirrhosis, there is a decrease in the proportion of CD4 + T-CCR6. Based on marker genes, CD4 + T-CCR6 is commonly associated with Th17 cells, primarily exerting its effects through the production of IL-17 family cytokines. CCR6, primarily expressed on Th17 cells and regulatory T cells (Tregs), functions as a chemokine receptor. And previous studies suggest that the CCR6 inhibits sepsis-induced liver injury through the CCR6-CCL20 axis (30).

In HBV cirrhosis, there is an increase in the proportion of CD8 + T-FABP5. FABP5, a fatty acid-binding protein, is responsible for various functions, including intracellular lipid metabolism and signal transduction. FABP5 may influence the survival, proliferation, and effector functions of CD8 + T cells by regulating intracellular lipid metabolism, thereby affecting its role in HBV cirrhosis (31).

### Exploring the distribution of immune cells in HBV and non-viral cirrhosis with spatial transcriptomics data

To investigate the spatial distribution of differential immune cell subclusters between HBV and non-viral cirrhosis, we utilized publicly available spatial transcriptomics samples from HBV cirrhosis (32). Using the Cell2location algorithm, we deconvoluted our single-cell data to determine the cellular composition of each spatial spot. We visualized the spatial distribution of immune cells (Figure 5I) and found Myeloid cells are colocalized with T cells, exhibiting an exclusionary distribution pattern in relation to NK cells in HBV cirrhosis. Following this, to explore differences in the co-localization of immune cells between HBV and non-viral cirrhosis, we analyzed cell barcode matrices using the Mistyr algorithm. Our analysis revealed that in HBV cirrhosis, myeloid cells are colocalized with T cells within a range of two spatial transcriptomic spots (Figure 5J).

## Discussion

**Figure 6:**
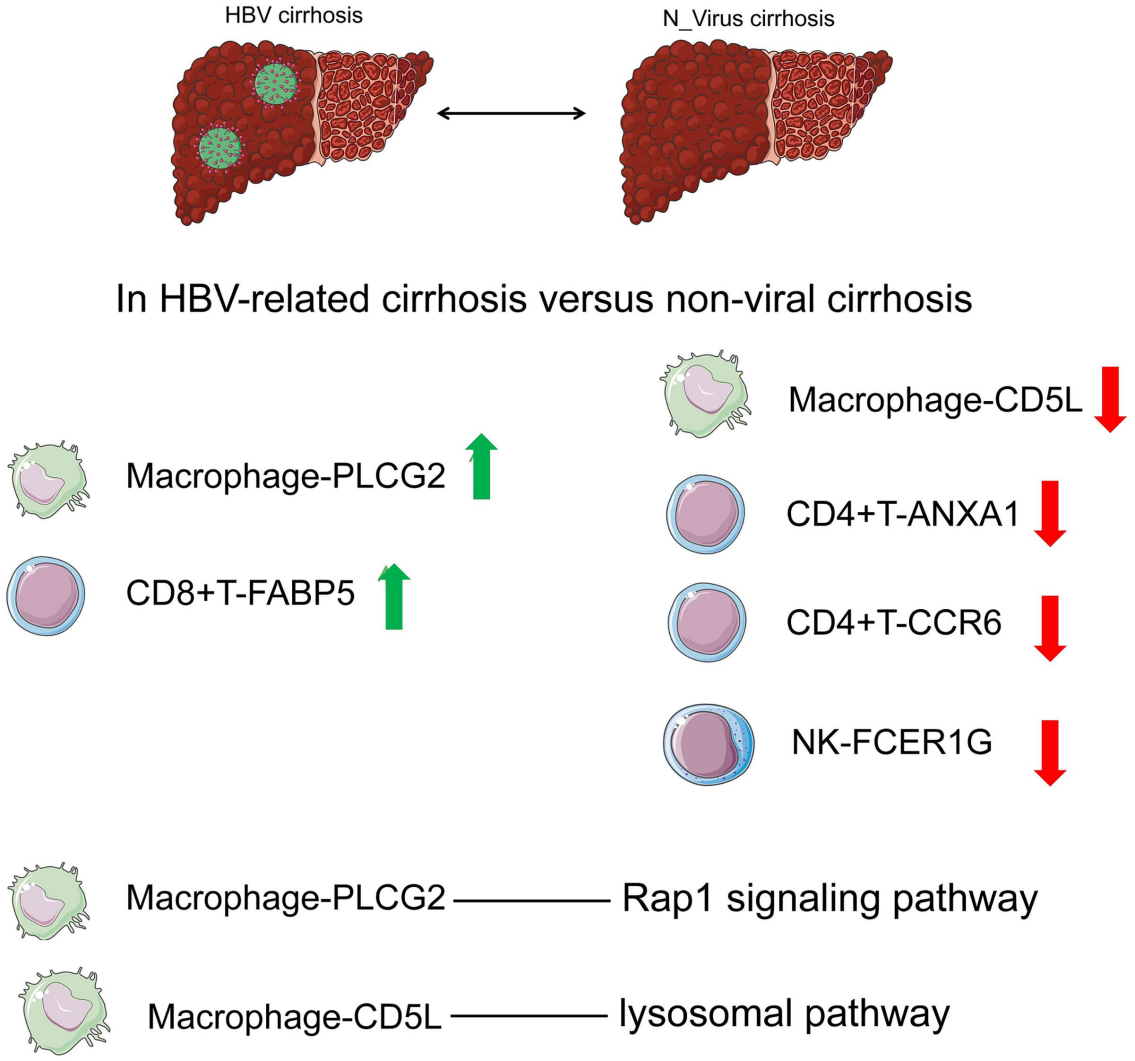
Comparison of immune cell changes between HBV cirrhosis and non-viral cirrhosis.

Our study delineates the immune heterogeneity between HBV and non-viral cirrhosis through a high-resolution, comparative single-cell analysis. The most distinctive findings center on the identification of a previously unappreciated immunological axis characterized by the expansion of Macrophage-PLCG2 and the reduction in Macrophage-CD5L, coupled with a concomitant reduction in cytotoxic NK-FCER1G cells in HBV cirrhosis (Figure 6A). This shift, along with the alteration in T cell subsets (e.g., increased CD8 + T-FABP5 and decreased CD4 + T-ANXA1/CCR6) (Figure 6A) and the discovery of unique cell–cell communication patterns such as the enhanced HLA-E/KLRK1 axis, suggests a remodeled immune landscape that may favor disease progression in HBV-related liver fibrosis.

In HBV infection, CD4 + and CD8 + T cells play crucial yet distinct roles in both controlling viral infection and mediating liver immunopathology. We identified an increased expression of CD8 + T-FABP5 in HBV cirrhosis. Studies have shown that FABP5 is highly expressed in exhausted CD8 + T cells within HCC tumor tissues rather than adjacent non-cancerous tissues. Additionally, this high expression of FABP5 in CD8 + T cells is positively correlated with recurrence-free survival in patients (33), suggesting that CD8 + T-FABP5 may represent a population of exhausted CD8 + T cells abundant in chronic hepatitis.

On the other hand, CD4 + T-ANXA1 and CD4 + T-CCR6 are decreased in HBV cirrhosis. HBV infection may lead to decreased expression of CD4 + T-ANXA1, resulting in reduced apoptosis of inflammatory cells and weakened anti-inflammatory responses, thereby promoting the development of cirrhosis. Collectively, these T cell subset changes suggest altered antiviral effector functions and impaired immunoregulation in HBV cirrhosis.

Macrophages play a crucial role in HBV infection. On one hand, they exert direct antiviral effects by producing soluble inflammatory mediators and other cytokines (such as Type I IFN, TNF-*α*, IL-6), which can directly inhibit HBV replication or induce damage or apoptosis of HBV-infected liver cells. On the other hand, they indirectly exert antiviral effects by synthesizing various cytokines and chemokines to activate or recruit anti-HBV inflammatory cells.

Conversely, HBV also induces the differentiation of liver macrophages. Macrophages are mainly classified into M1 (pro-inflammatory) and M2 (anti-inflammatory and repair) types. It has been observed that in patients with chronic HBV infection accompanied by liver fibrosis, there is an increase in M2 macrophages, with M2 macrophages being predominantly expressed in the liver (34). Meanwhile, liver macrophages promote the differentiation of hepatic stellate cells into collagen-producing myofibroblasts, leading to collagen deposition and scar formation, which contributes to the development of liver fibrosis (35).

Our research findings indicate a close association between Macrophage-PLCG2 and the progression of HBV cirrhosis (amplification of Macrophage-PLCG2 in HBV cirrhosis). PLCG2, also known as phospholipase Cγ2, is involved in macrophage signaling transduction, thereby regulating macrophage function. PLCG2 was found to contribute greatly to the immunosuppressive microenvironment and enhanced immune escape (36).

Pathway enrichment analysis revealed associations with monocyte differentiation, RNA splicing, lymphocyte differentiation, and the C-type lectin receptor signaling pathway, as well as the TNF signaling pathway, suggesting that this macrophage subset may originate from monocytes and may directly inhibit HBV by producing TNF cytokines.

Interestingly, Macrophage-CD5L is decreased in HBV cirrhosis. CD5L, also known as Apoptosis Inhibitor of Macrophage (AIM), is a protein produced by tissue macrophages that regulates macrophage activity and participates in the occurrence of infection and inflammation. In the liver, CD5L can activate hepatic stellate cells, leading to liver fibrosis (37). Moreover, it is believed to promote recovery from many diseases. CD5L can also promote macrophage polarization toward the M2 phenotype by enhancing autophagy, thereby exerting immunosuppressive effects (38). However, the role of CD5L in HBV infection is not yet fully understood and requires further research. Together, the expansion of Macrophage-PLCG2 and reduction of Macrophage-CD5L indicate a shift in macrophage polarization states in HBV cirrhosis.

NK cells, as crucial innate immune cells, rapidly identify and eliminate virus-infected liver cells. Under normal circumstances, NK cells secrete IFN-*γ*, which suppresses the activation of hepatic stellate cells by reducing profibrogenic TGF-*β* signaling, inducing apoptosis of activated stellate cells, and ultimately inhibiting liver fibrosis (39, 40). In HBV infection, HBV-induced suppressive monocytes induce the differentiation of NK cells into NK regulatory cells (NK-reg), resulting in decreased secretion of IFN-γ, increased IL-10, and accelerated development of cirrhosis (41). Our research also found a decreased expression of NK-FCER1G in HBV cirrhosis, possibly due to the inhibitory effect of high levels of IL-10 on NK cell expression and function during HBV infection (42). FCER1G is a protein expressed on the surface of immune cells such as eosinophils, dendritic cells, and NK cells. Further research is needed in the field of HBV and cirrhosis regarding the role of NK-FCER1G. The expression of NK-FCER1G is associated with negative regulation of monocyte differentiation, lymphocyte differentiation, RNA splicing, myeloid cell differentiation, and phosphate metabolism processes (24). These findings point to impaired NK cell cytotoxicity and potential dysfunction in HBV cirrhosis.

Our study provides new insights into immune dysregulation in cirrhosis, though several limitations should be noted. Firstly, due to the difficulty in collecting samples, we included public single-cell data on cirrhosis. Additionally, since we did not conduct spatial transcriptomics on cirrhosis samples and there are currently no publicly available spatial transcriptomics data for cirrhosis, we used samples from normal regions of liver cancer patients with HBV cirrhosis for our analysis. Thirdly, we did not include neutrophils in our analysis because they are easily destroyed during cell isolation. In future studies, we aim to optimize our methods to gain a clearer understanding of the role of neutrophils in cirrhosis. Finally, our spatial transcriptomic analysis utilized data from the non-tumorous liver tissue of HBV-HCC patients to infer spatial patterns in cirrhosis. While this approach is justified given the shared etiology and the common presence of underlying cirrhosis in these peri-tumoral samples, we acknowledge this as a limitation. The immune landscape in the immediate vicinity of a tumor is known to be altered by tumor-derived factors, a phenomenon known as the field effect. Consequently, the myeloid-T cell colocalization and enhanced HLA-E/KLRK1 signaling we observed might be influenced by the pro-tumoral microenvironment. Nonetheless, these findings provide crucial initial insights into the spatial organization of immune cells in HBV-damaged livers and highlight patterns that may be relevant for both cirrhosis and subsequent carcinogenesis. Future studies utilizing data on pure cirrhosis samples will be essential to validate these observations.

Beyond characterizing immune heterogeneity, our study identifies several findings with potential translational relevance. The specific cell subpopulations and signaling pathways identified hold promise for the development of novel therapeutic strategies and biomarkers for HBV-related liver disease. First, the Macrophage-PLCG2 subset, which exhibits an anti-inflammatory phenotype and is expanded in HBV cirrhosis, could represent a novel therapeutic target. Given its association with immunosuppressive functions, targeting PLCG2 signaling might help to rebalance the immune microenvironment, potentially slowing the progression of fibrosis or preventing carcinogenesis. Conversely, the observed reduction of NK-FCER1G cells in HBV cirrhosis suggests that strategies to expand or activate this NK cell subset might restore effective immune surveillance. Second, the enhanced HLA-E/KLRK1 (NKG2D) signaling axis between myeloid and NK cells presents a compelling opportunity for immunotherapeutic intervention. Agonists of this pathway, such as NKG2D-ligand mimetics, could be explored to boost the cytotoxic function of NK cells against HBV-infected hepatocytes or pre-malignant cells in the cirrhotic liver. Additionally, the aberrant colocalization of myeloid and T cells, along with the enhanced HLA-E/KLRK1 signaling noted earlier, suggests complex immune cell interactions that could be targeted therapeutically. From a diagnostic standpoint, the distinct signatures of these cell subsets could be leveraged for biomarker discovery. Measuring the abundance or transcriptional activity of Macrophage-PLCG2 or CD8 + T-FABP5 in peripheral blood mononuclear cells or through non-invasive liquid biopsy approaches might help in stratifying patients based on their risk of progression to decompensated cirrhosis or HCC. This would enable more personalized monitoring and early intervention.

In conclusion, through an integrated analysis of single-cell transcriptomic data, we have delineated distinct immune cell landscapes in HBV-related versus non-viral cirrhosis. Our findings highlight the expansion of anti-inflammatory Macrophage-PLCG2 and CD8 + T-FABP5 subsets in HBV cirrhosis, alongside diminished NK-FCER1G and altered T cell populations, suggesting a remodeled immune microenvironment that may contribute to disease progression. It should be noted that the initial clinical cohort was limited in size, and the conclusions drawn, while supported by integration with public datasets, warrant further validation in larger, independent patient cohorts. Nonetheless, this study provides a foundational resource for understanding immune dysregulation in HBV cirrhosis and identifies potential cellular targets for future therapeutic exploration.

## Data Availability

Publicly available datasets were analyzed in this study. This data can be found here: Single-cell RNA sequencing raw data published in NCBI (RPRJNA833766).
